# Supplementary figures and images for: The Effect of Anthocyanins from Dioscorea alata L. on Antioxidant Properties of Perinatal Hainan Black Goats and Its Possible Mechanism in the Mammary Gland
Source: Animals (Basel). 2022 Nov 28;12(23):3320. doi: 10.3390/ani12233320 (PMC9735849; doi:10.3390/ani12233320)

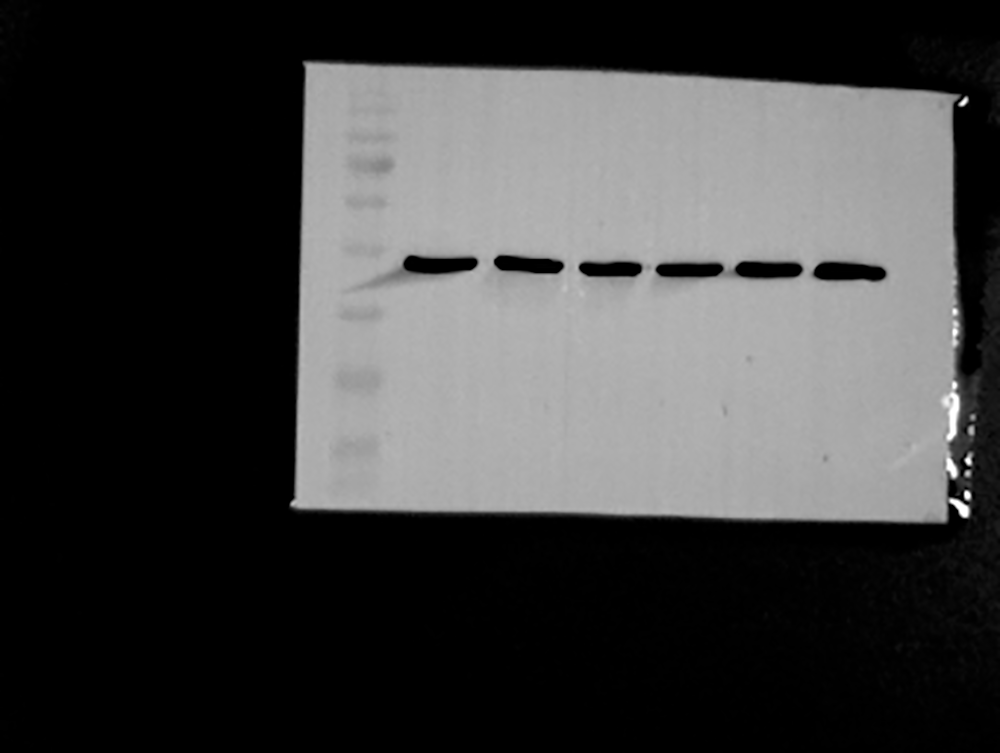

Supplement: Supplementary file 1 [file animals-12-03320-s001.zip › Supplementary Figure S1 a┬-Actin.png]

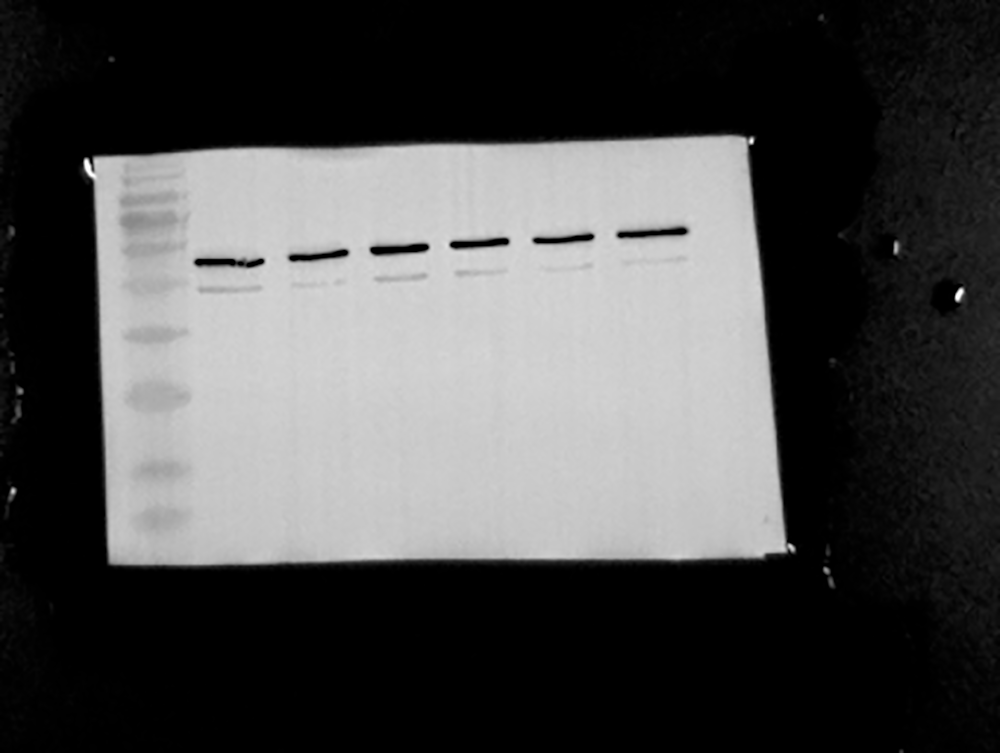

Supplement: Supplementary file 1 [file animals-12-03320-s001.zip › Supplementary Figure S2 JNK.png]

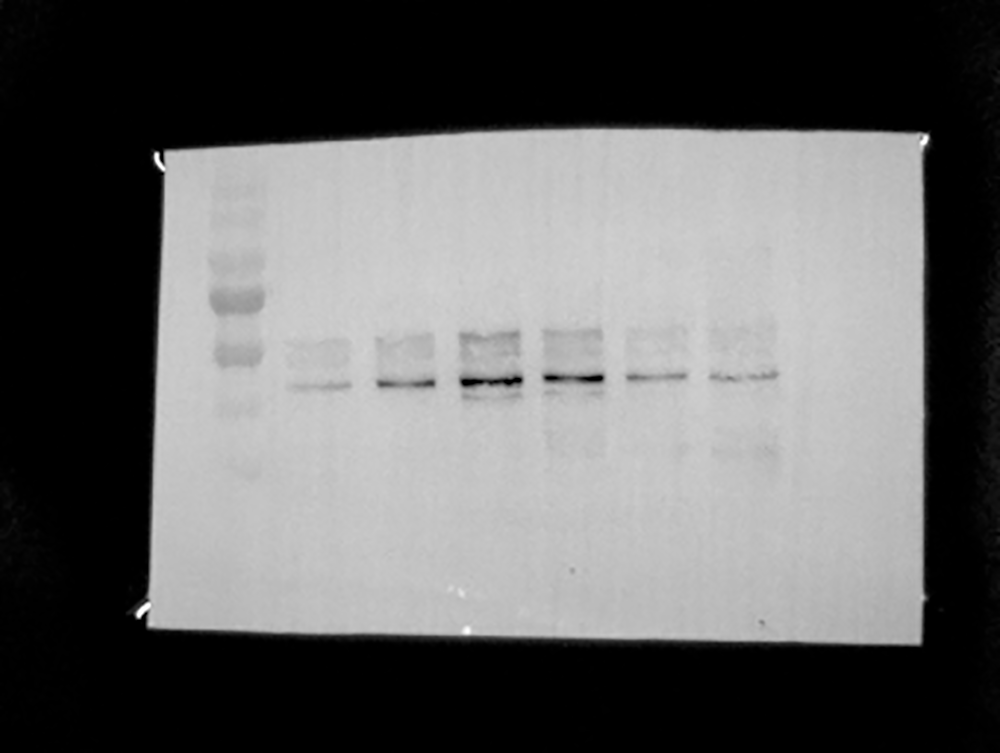

Supplement: Supplementary file 1 [file animals-12-03320-s001.zip › Supplementary Figure S3 P-JNK.png]

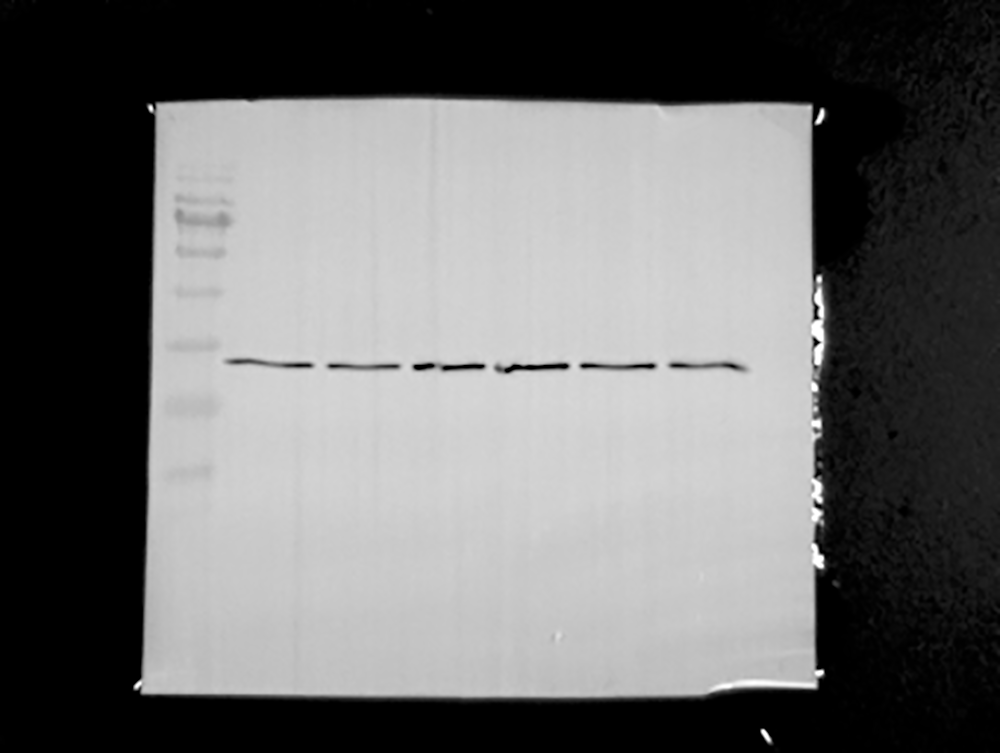

Supplement: Supplementary file 1 [file animals-12-03320-s001.zip › Supplementary Figure S4 Caspase-3.png]

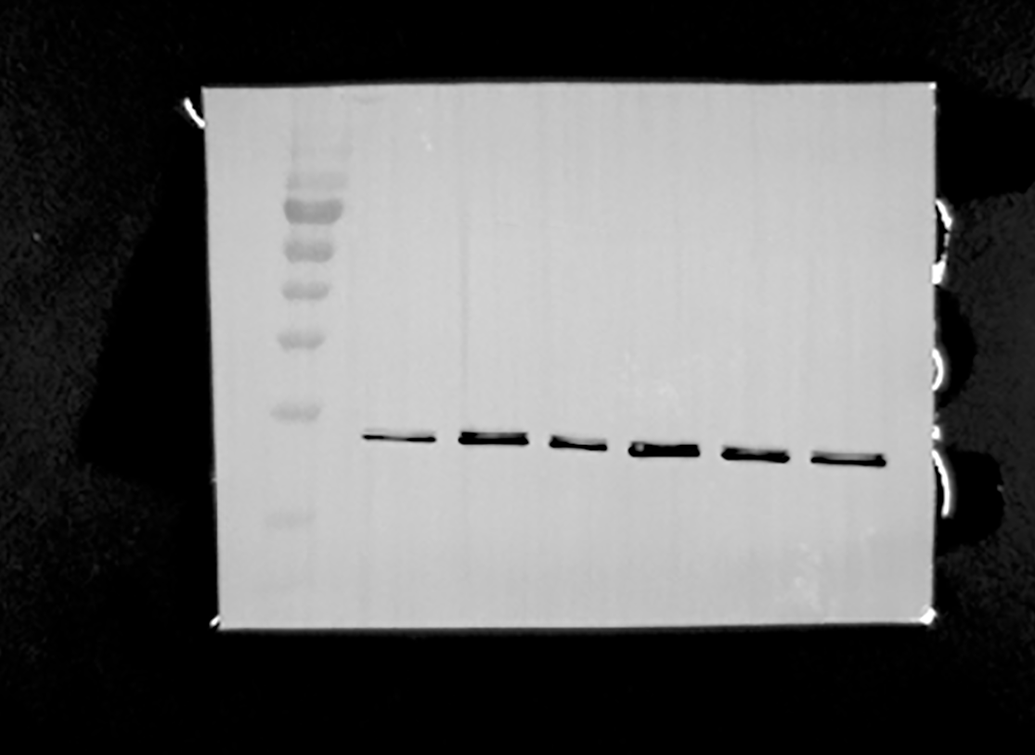

Supplement: Supplementary file 1 [file animals-12-03320-s001.zip › Supplementary Figure S5 Cytochrome C.png]
